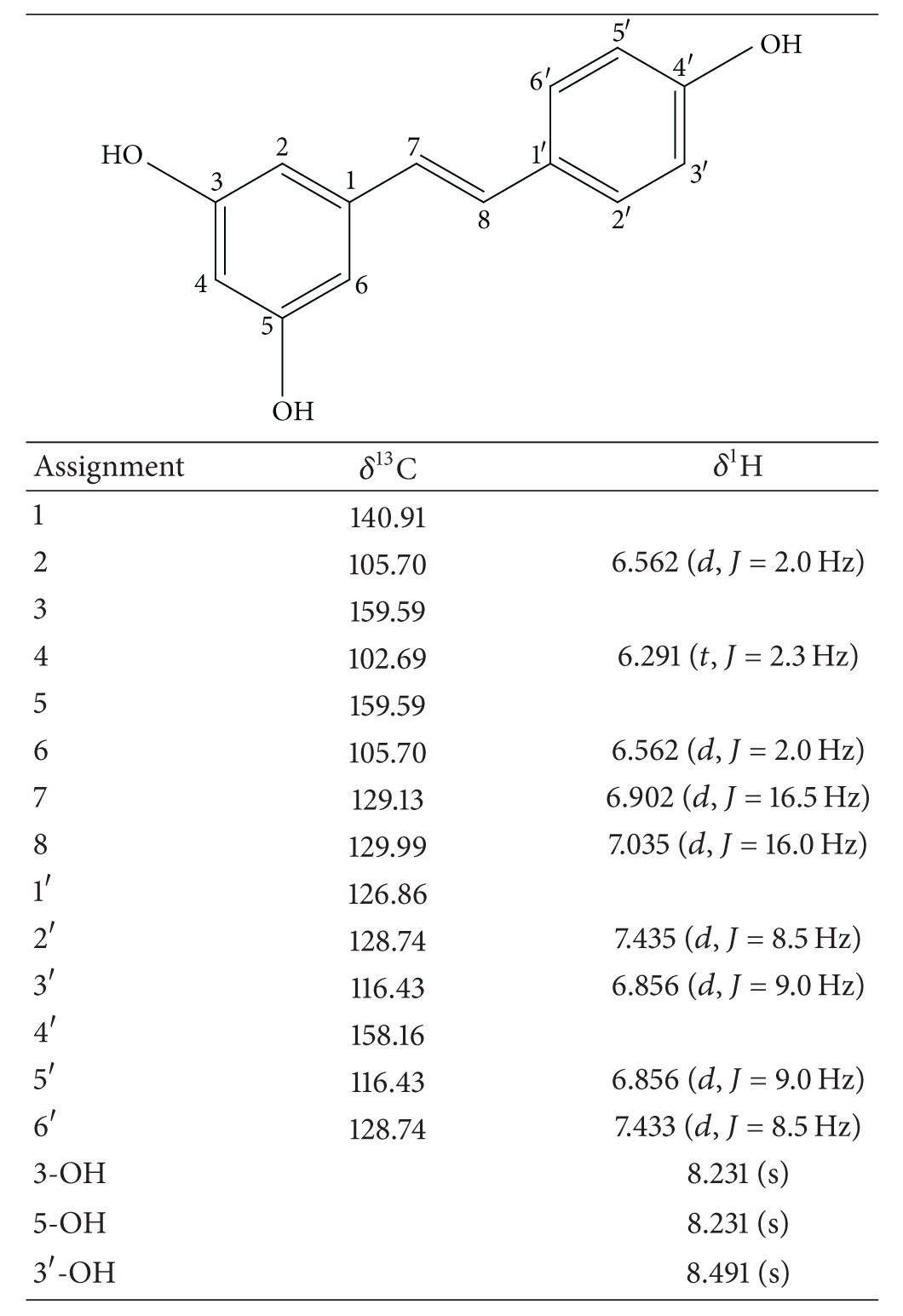# Isolation of Resveratrol from Vitis Viniferae Caulis and Its Potent Inhibition of Human Tyrosinase

**DOI:** 10.1155/2013/645257

**Published:** 2013-02-12

**Authors:** Jiaa Park, Yong Chool Boo

**Affiliations:** BK21 Medical Education Program for Human Resources, Department of Molecular Medicine and Cell and Matrix Research Institute, Kyungpook National University School of Medicine, Jung-gu, Daegu 700-422, Republic of Korea

## Abstract

Tyrosinase (TYR) catalyzes rate-limiting reactions of cellular melanin synthesis, and its inhibitors are of commercial interest as potential skin whitening agents. However, the limited availability of human TYR makes the screening of TYR inhibitors difficult. To overcome this hurdle, we transformed nonmelanocytic human embryonic kidney (HEK) 293 cells to express human TYR constitutively. Using these cells as a source of human TYR, the ethanolic extracts of 52 medicinal plants grown in Korea were tested for human TYR activity, and the extract of Vitis Viniferae Caulis (dried stems of the grape tree, *Vitis vinifera* L.) was found to inhibit human TYR activity potently. An active compound was isolated from this extract by solvent fractionation followed by liquid column chromatography and identified as resveratrol by spectroscopic and chromatographic analyses. Resveratrol was determined to be a highly potent inhibitor of human TYR (IC50 = 0.39 **μ**g mL^−1^) as compared with p-coumaric acid (IC50 = 0.66 **μ**g mL^−1^) and arbutin (IC50 > 100 **μ**g mL^−1^) and inhibited melanin synthesis by human epidermal melanocytes at subtoxic concentrations. This study suggests that resveratrol and resveratrol-containing extracts of Vitis Viniferae Caulis have a potential use as skin whitening agents.

## 1. Introduction

Skin color is a major contributor to human beauty and attractiveness [1]. The color of skin is determined by the composition and distribution of various chromophores, such as melanin, hemoglobin, bilirubin, and carotenoids. Melanin is the major dark pigment found in skin, hair, and eyes and provides protection against harmful ultraviolet radiation that can cause photoageing and photocarcinogenesis [2]. In fact, the incidence of skin cancer was reduced by artificially enhancing melanin synthesis in mice [3]. Furthermore, the frequency of malignant melanoma is lower in dark-skinned people [4]. However, excessive melanin deposition can cause aesthetic skin problems, such as melasma, freckles, and senile lentigines. The cosmetic relevance of melanin in skin has prompted the research and development of cosmeceuticals that inhibit melanin synthesis. Although various melanogenesis inhibitors, such as arbutin, have been incorporated into cosmetics to control unwanted skin pigmentation [5, 6], their efficacies remain controversial.

Melanocytes at the stratum basale of the epidermis synthesize melanin [7], and a group of melanogenic enzymes are involved in the synthesis of melanin in melanosomes [8]. Tyrosinase (TYR, monophenol, dihydroxyphenylalanine:oxygen oxidoreductase, EC 1.14.18.1) catalyzes the initial oxidations of L-tyrosine and L-3,4-dihydroxyphenylalanine (DOPA) to DOPA quinone, which conjugates with cysteine or glutathione to produce 5-S-cysteinyl DOPA and glutathionyl DOPA, respectively, and these conjugates in turn are progressively polymerized to the reddish-yellow pheomelanin. The oxidation of DOPA quinone to DOPA chrome without conjugation to thiol compounds leads to the synthesis of the brownish-black eumelanin. Melanin-carrying melanosomes are then transferred via dendrites to neighboring keratinocytes in the epidermis [9]. Because TYR is the rate-limiting enzyme of the melanogenic pathway, it has been a major target for the control of unwanted skin pigmentation, and as a result, numerous agents have been found to inhibit TYR expression or activity [5, 6, 10–12]. 

In many previous studies, presumably because of availability problems, mushroom TYR has been used instead of human TYR, for the screening of potential skin whitening agents [13, 14]. However, this approach is problematic because the biochemical properties of human and mushroom TYRs are quite different [15, 16]. In particular, many compounds inhibit these enzymes to markedly different extents [17, 18], and therefore, human TYR is essential for accurate screening. To meet this demand, we recently established a human embryonic kidney (HEK) 293-TYR cell line to provide human TYR. This cell line was generated by the stable transfection of nonmelanocytic HEK293 cells with a plasmid that encodes human TYR [19]. These transformed cells proliferated rapidly and expressed the active form of human TYR constitutively. Furthermore, lysates of these cells were used successfully in our recent studies [20].

The aims of the present study were to screen for plant extracts that inhibit human TYR and to isolate and identify the compound in the selected extract. Taking advantage of the established HEK293-TYR cell line as a source of human TYR, extracts of various medicinal plants grown in Korea were subjected to primary screening assays against human TYR. Of the 52 plant extracts tested, the extract of Vitis Viniferae Caulis (VVC) was found to inhibit human TYR activity potently. Thus, this study was focused on VVC extract. 

## 2. Materials and Methods

### 2.1. Plant Extracts

Ethanolic extracts of medicinal plants used in the initial screening assays were purchased from the Plant Extract Bank of Korea (Daejeon, Republic of Korea) (http://extract.pdrc.re.kr/). Yields of extracts are shown in Table 1.

### 2.2. Purification and Identification of the Active Compound X (Resveratrol) in VVC

VVC (dried stems of the grape tree, *Vitis vinifera* L.) was purchased from Jayeoncho (Seoul, Republic of Korea) (http://www.jherb.com/) and extracted in our laboratory. Plant material (200 g) was ground and extracted with 95% (v/v) aqueous ethanol at room temperature. The solution so-obtained was then evaporated under reduced pressure to dryness to produce VVC extract (yield: 4.8%).

VVC extract (8 g) was suspended in water and liquid-liquid partitioned successively with methylene chloride (MC), ethyl acetate (EtOAc), and n-butanol (BuOH), and the solvent fractions obtained were evaporated to dryness. Fraction yields were MC fraction 23%, EtOAc fraction 12% BuOH fraction 9%, and aqueous (H_2_O) fraction 56%. The EtOAc fraction inhibited human TYR activity most potently.

The EtOAc fraction (2 mg) was dissolved in ethanol and applied to a silica-coated TLC plate (Merck, Darmstadt, Germany) as a long streak. Ascending TLC was run using a mixture of chloroform, methanol, and acetic acid (90 : 10 : 1, v/v/v) as a mobile phase in an airtight container. Separated bands were viewed under a UV lamp. The TLC plate was then divided into 11 horizontal sectors, and the silica of each sector was scraped into 100 *μ*L of 75% ethanol, vortexed, and centrifuged. Supernatants (4 *μ*L) were subjected to TYR assay, and the compound found to inhibit TYR was designated compound X.

The EtOAc fraction (0.9 g) was then subjected to column chromatography to purify compound X. First separation was performed on a Sephadex LH20 column (Pharmacia Fine Chemical Co., Ltd., Uppsala, Sweden) (3 cm × 20 cm) by eluting with MC : methanol (1 : 1). The eluate was monitored at 280 nm and analyzed by TLC. Fractions containing compound X were pooled and evaporated to dryness. This material (0.3 g) was further separated on a YMC-gel ODS-A column (YMC Co. Ltd., Kyoto, Japan) (3 cm × 20 cm) by step gradient elution using 20~60% aqueous methanol. The eluate was monitored at 280 nm and analyzed by TLC. Compound X-containing fractions were combined and evaporated to yield an amorphous powder (52 mg).

### 2.3. Instrumental Analyses

UV spectrum was recorded on a Shimadzu UV-1650PC spectrophotometer (Shimadzu Corporation, Kyoto, Japan). NMR spectrum was recorded using a 500 MHz Varian Unity INOVA 500 FT-NMR spectrometer (Varian, Inc., Palo Alto, CA, USA) at room temperature. Chemical shifts (*δ* ppm) are reported relative to tetramethylsilane. HPLC analysis was done using a Gilson HPLC system (Gilson, Inc. Middleton, WI, USA) equipped with a 321 pump and UV/VIS 151 detector. Separation was done on a 5 *μ*m Hector-M C18 column (4.6 mm × 250 mm) (RStech Co. Daejeon, Republic of Korea). The mobile phase consisted of 0.5% formic acid (A) and acetonitrile (B). The gradient was programmed as follows: 0–50 min, a linear gradient from 30 to 100% B; 50–65 min, 100% B; 65–70 min, a linear gradient from 100 to 30% B; 70–80 min, 30% B. The flow rate was 0.6 mL min^−1^. The UV detector was set at 280 nm. Calibration curves were generated using authentic resveratrol purchased from Sigma-Aldrich (St. Louis, MO, USA). 

### 2.4. Cell Culture and Preparation of Cell Lysates

Human epidermal melanocytes (HEMs), derived from moderately pigmented neonatal foreskins, were obtained from Cascade Biologics (Portland, OR, USA). Cells were grown in Medium 254 supplemented with human melanocyte growth supplement (Cascade Biologics) and antibiotics. HEK293 cells and murine melanoma B16/F10 cells (CRL-6475) were obtained from the American Type Culture Collection (Manassas, VA, USA). HEK 293 cells were transformed into HEK293-TYR cells stably expressing human TYR, as previously described [19]. HEK293 cells, HEK293-TYR cells, and murine melanoma B16/F10 cells were cultured in growth medium (Dulbecco's minimum Eagle's medium (DMEM) containing 10% fetal bovine serum and antibiotics (100 U mL^−1^ penicillin, 0.1 mg mL^−1^ streptomycin, and 0.25 *µ*g mL^−1^ amphotericin B)). Cells were cultured at 37°C in a humidified atmosphere containing 5% CO_2_ and 95% air. Cells were lysed in ice-cold lysis buffer (10 mM Tris-Cl, pH 7.4, 120 mM NaCl, 25 mM KCl, 2.0 mM EGTA, 1.0 mM EDTA, and 0.5% Triton X-100 and protease inhibitor cocktail) and centrifuged at 13,000 ×g for 15 min at 4°C to obtain clear supernatants which were used as cell lysates.

### 2.5. Assay for TYR Activity

*In vitro* TYR assays were run for human, murine, and mushroom TYRs. The lysates of HEK293-TYR cells were used as a human TYR preparation in most experiments. In some experiments, the lysates of murine B16/F10 cells (Figure 5), HEK293 cells, and HEMs (Figure 6) were used for comparative purposes. Mushroom TYR was purchased from Sigma-Aldrich. The reaction mixture (200 *μ*L) containing either cell lysates (40 *μ*g protein) or mushroom TYR (20 U) and 0.5 mM L-tyrosine, 1 *μ*M DOPA in 100 mM sodium phosphate buffer (pH 6.8) was placed on a 96-well microplate and incubated at 37°C. The formation of DOPA chrome was monitored by measuring absorbance at 490 nm with a BioRad Model 680 microplate reader (Bio-Rad Laboratories, Inc., Hercules, CA, USA). Assays were also run in the absence of TYR enzyme to correct for potential nonenzymatic reactions. Samples and reference inhibitors were included in reaction mixtures at different concentrations.

### 2.6. Western Blotting

Western blotting of the cell lysates was conducted under a denatured condition as previously described [19, 21]. Primary antibodies for TYR, tyrosinase-related protein 1 (TRP1), tyrosinase-related protein 2 (TRP2), and glyceraldehyde 3-phosphate dehydrogenase (GAPDH) were purchased from Santa Cruz Biotechnology (Santa Cruz, CA, USA). Immunoreactive bands were detected using a picoEPD Western Reagent kit (ELPIS-Biotech, Daejeon, Republic of Korea). 

### 2.7. Assay for Melanin Synthesis in HEMs

Melanogenesis in HEMs was determined as previously described [19]. Cells were pretreated with test material for 60 min, and then melanogenesis was stimulated by adding L-tyrosine (1.0 mM). Media were replaced every other day, and cells were cultured for 6 days. Intracellular melanin was extracted using 0.1 M NaOH at 60°C for 60 min, quantified by measuring absorbance at 490 nm, and normalized for protein content using the Bio-Rad DC assay. Cell viabilities were determined using 3-[4,5-dimethylthiazol-2-yl]-2,5-diphenyltetrazolium bromide.

### 2.8. Statistical Analysis

Results are presented as the mean ± SE of three or more independent experiments. Significant differences between groups were determined by one-way ANOVA at *P* values of <0.05.

## 3. Results

**Figure 1:**
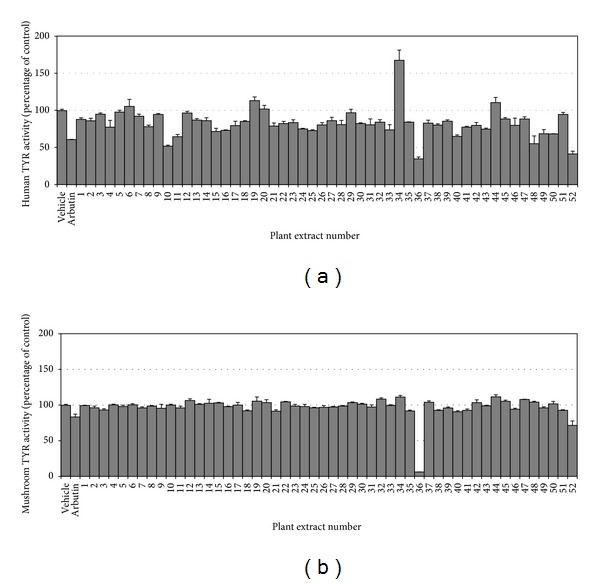
Effects of plant extracts on the activities of human and mushroom TYRs. The activities of human TYR (a) and mushroom TYR (b) were determined in the absence or presence of plant extract (100 *µ*g mL^−1^) or arbutin (80 *µ*g mL^−1^). Data are presented as percentages of uninhibited activities (means ± SE, *n* = 3). *P < 0.05 versus vehicle control.

The effects of various plant extracts on human TYR activity were determined *in vitro* using HEK293-TYR cell lysates. The plant extracts tested in the present study are listed in Table 1. In this assay, 100 *µ*g mL^−1^ of each plant extract was tested. Arbutin was used as a positive reference compound at a concentration of 80 *µ*g mL^−1^. Of the 52 plant extracts tested, the strongest inhibition of human TYR was exhibited by the extract of Mori Ramulus (number 36) and the next strongest was the extract of VVC (number 52) (Figure 1(a)). For comparative purposes, the effects of the 52 plant extracts on mushroom TYR were also examined (Figure 1(b)). Mori Ramulus extract inhibited mushroom TYR activity strongly, but the other plant extracts, including VVC extract, did not exhibit remarkable inhibition. These results indicate that the extract of VVC contains constituents that preferentially inhibit human TYR. Thus, VVC extract was examined further.

**Figure 2:**
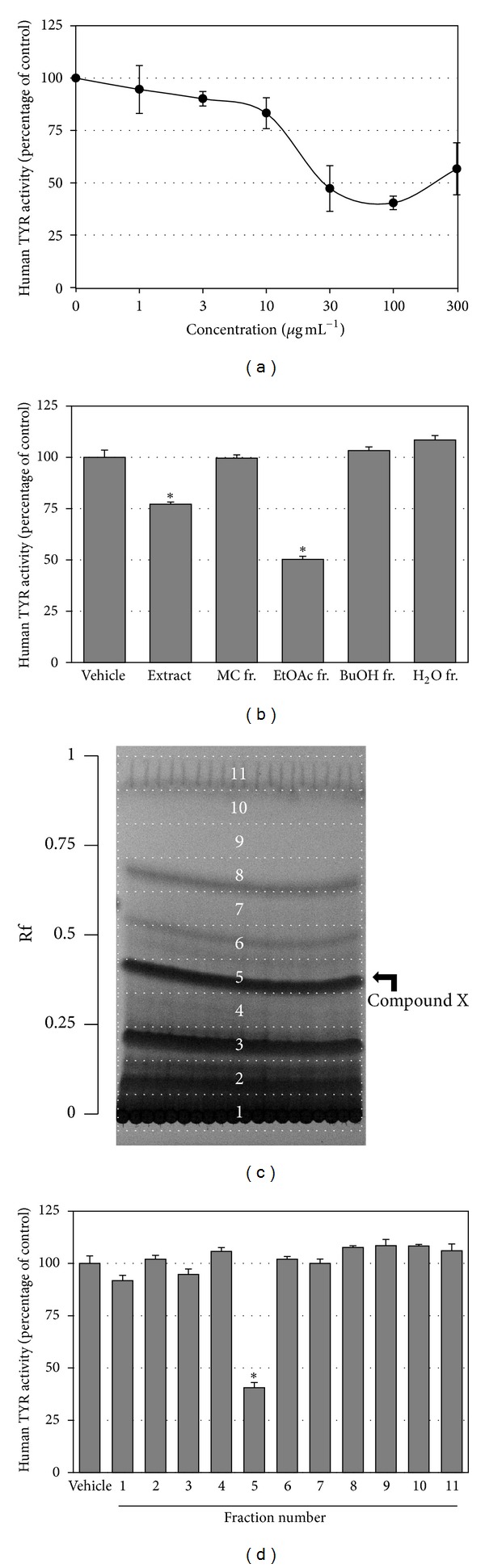
Analysis of constituents of VVC responsible for the inhibition of human TYR activity. VVC extract was tested against human TYR activity at different concentrations (a). VVC extract was separated into four different solvent fractions and tested against human TYR activity at 10 *µ*g mL^−1^ (b). The EtOAc fraction was further separated into 11 subfractions by preparatory TLC (c) and tested against human TYR activity (d). The major band in number 5 fraction was presumed to be an active constituent and denoted compound X. Data are presented as percentages of uninhibited activities (means ± SE, *n* = 3). *P < 0.05 versus vehicle control.

In the next experiment, the VVC extract was tested at different concentrations. As shown in Figure 2(a), the IC50 value of VVC extract was approximately 30 *µ*g mL^−1^, indicating that the extract itself is a more potent inhibitor of human TYR than arbutin. VVC extract produced turbidity at higher concentrations, making the assay inaccurate.

VVC extract was liquid-liquid partitioned, and solvent fractions were tested for human TYR activity at 10 *µ*g mL^−1^ (Figure 2(b)). Of the different fractions (MC, EtOAc, BuOH, and aqueous), only the EtOAc fraction exhibited inhibitory effects against human TYR (IC50 = 10 *µ*g mL^−1^), indicating the active compounds were enriched in this fraction. Thus, the EtOAc fraction was further separated by preparatory TLC (Figure 2(c)), and the resulting 11 subfractions were tested for human TYR activity (Figure 2(d)). The results obtained indicated that number 5 fraction most potently inhibited human TYR activity. The major band in this fraction was denoted compound X.

**Figure 3:**
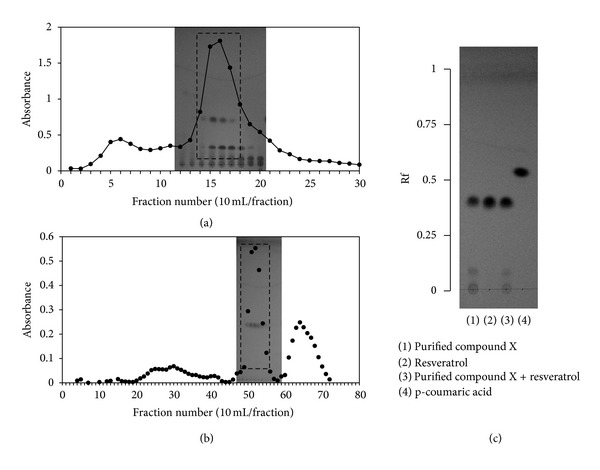
Purification of compound X and its identification as resveratrol. The EtOAc fraction derived from VVC extract was separated on a Sephadex LH20 column (a) And the elution fractions were analyzed by TLC for compound X. Compound-X-containing fractions were then pooled, concentrated, and subjected to second chromatography on an ODS-A column (b). Elution fractions containing compound X were combined and evaporated to obtain purified compound X, which was compared with commercially sourced resveratrol by TLC (c). Ascending TLC was run on a silica-coated plate using a mixture of chloroform, methanol, and acetic acid (90 : 10 : 1, v/v/v) as the mobile phase.

**Figure 4:**
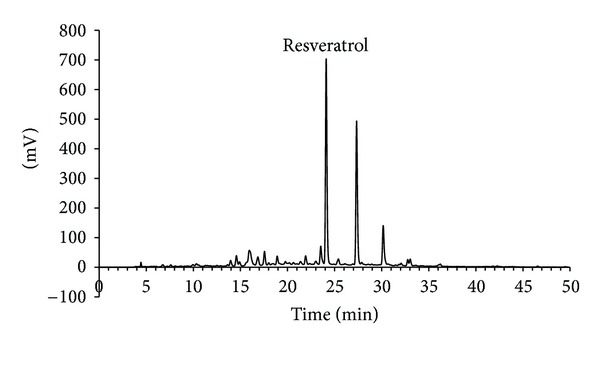
Fingerprint HPLC of VVC extract. VVC extract was analyzed by HPLC as described in Section 2. The chromatogram at 280 nm is shown. The peak of resveratrol was assigned by cochromatography with authentic resveratrol.

In the next step, compound X was purified from the EtOAc fraction by successive column chromatography on Sephadex LH20 (Figure 3(a)) and ODS-A columns (Figure 3(b)). Elution fractions were monitored by UV absorbance and analyzed by TLC to check for the presence of compound X. Purified compound X was subjected to NMR analysis. ^1^H- and ^13^C-NMR spectroscopic data are provided in Table 2. Based on these data and comparison with literature values [22], compound X was identified to be resveratrol. Cochromatography of purified compound X and authentic resveratrol on a TLC plate further verified this identification (Figure 3(c)). A typical HPLC pattern of VVC extract is shown in Figure 4. The content of resveratrol in VVC extract was determined to be 2.1%, indicating that VVC is a rich source of resveratrol. 

**Figure 5:**
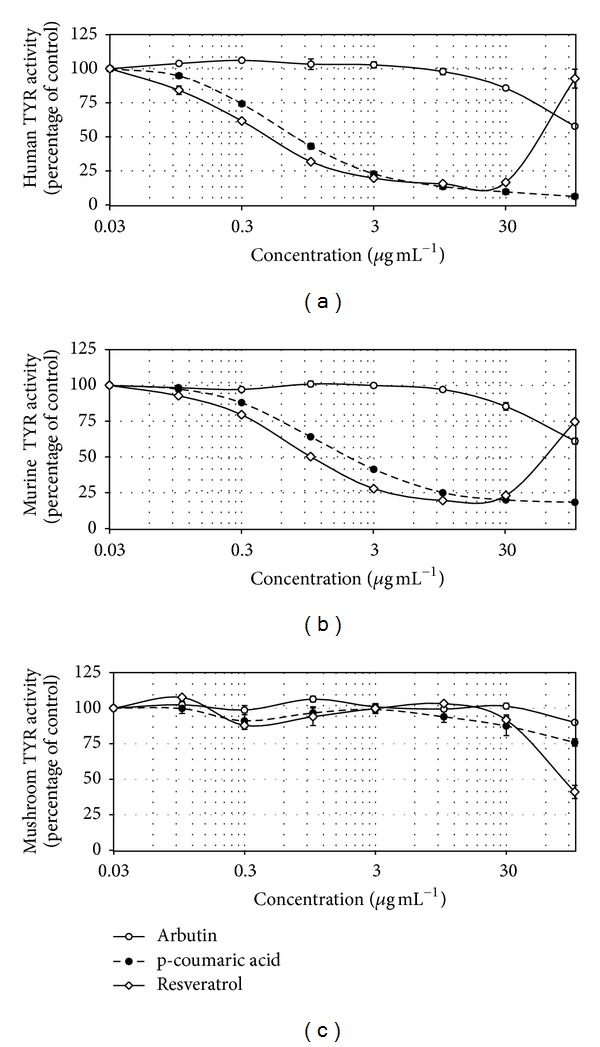
Effects of resveratrol, p-coumaric acid, and arbutin on the activities of TYRs of different origins. The activities of human TYR (a), murine TYR (b), and mushroom TYR (c) were determined in the absence or presence of test material at the indicated concentrations. Data are presented as percentages of uninhibited activities (means ± SE, *n* = 3).

The effects of resveratrol on human, murine, and mushroom TYRs were then examined and compared with those of arbutin and p-coumaric acid. p-Coumaric acid has been known as a potent inhibitor of human TYR and of cellular melanogenesis [18–20, 23]. In the assays using cell lysates, resveratrol generated an abnormal dose-response curve at high concentrations due to its property producing turbidity in the reaction mixture. However, its IC50 values were accurately determined at low concentration ranges. As shown in Figure 5(a), resveratrol inhibited human TYR activity more strongly (IC50 = 0.39 *µ*g mL^−1^) than p-coumaric acid (IC50 = 0.66 *µ*g mL^−1^) and arbutin (IC50 > 100 *µ*g mL^−1^). Resveratrol also inhibited murine TYR activity (IC50 = 1.0 *µ*g mL^−1^) more strongly than p-coumaric acid (IC50 = 2.0 *µ*g mL^−1^) and arbutin (IC50 > 100 *µ*g mL^−1^) (Figure 5(b)). Resveratrol had much smaller effect on mushroom TYR activity (Figure 5(c)) than on human and murine TYRs (Figures 5(a) and 5(b)).

**Figure 6:**
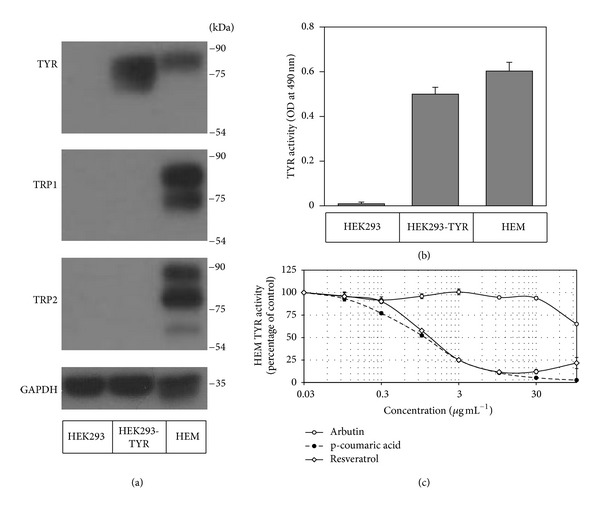
Effects of resveratrol, p-coumaric acid, and arbutin on human TYR activity determined using the lysates of HEMs. The lysates of HEK293 cells, HEK293-TYR cells, and HEMs were subjected to western blotting for TYR, TRP1, TRP2, and GAPDH (a) and *in vitro* TYR activity assay (b). The lysates of HEMs were used in the human TYR activity assay (c). Data are presented as percentages of uninhibited activities (means ± SE, *n* = 3).

TYR protein synthesized as a *de novo* form goes through an extensive process of posttranslational modifications to reach a fully matured active form in HEMs [24]. For example, N-glycosylation of TYR is critical for protein folding and activity [25]. To examine whether the TYR expressed in nonmelanocytic HEK293-TYR cells can be fully matured, the lysates of HEK293-TYR cells and HEMs were compared by western blotting and activity assay. As expected, HEK293-TYR cells expressed TYR, but not TRP1 and TRP2, whereas HEMs expressed all these melanogenic enzymes (Figure 6(a)). The TYR expressed in HEMs appeared mostly as the fully mature form (~80 kDa), and the TYR expressed in HEK293-TYR cells appeared as a mixture of mature and immature forms (Figure 6(a)). The TYRs expressed in HEK293-TYR and HEMs exhibited similar catalytic activities (Figure 6(b)). In addition, each of resveratrol, p-coumaric acid, and arbutin exhibited similar inhibition patterns against TYRs expressed in those two different cell types (Figures 5(a) and 6(c)). These results support the molecular similarity between TYRs expressed in HEK293-TYR cells and HEMs.

**Figure 7:**
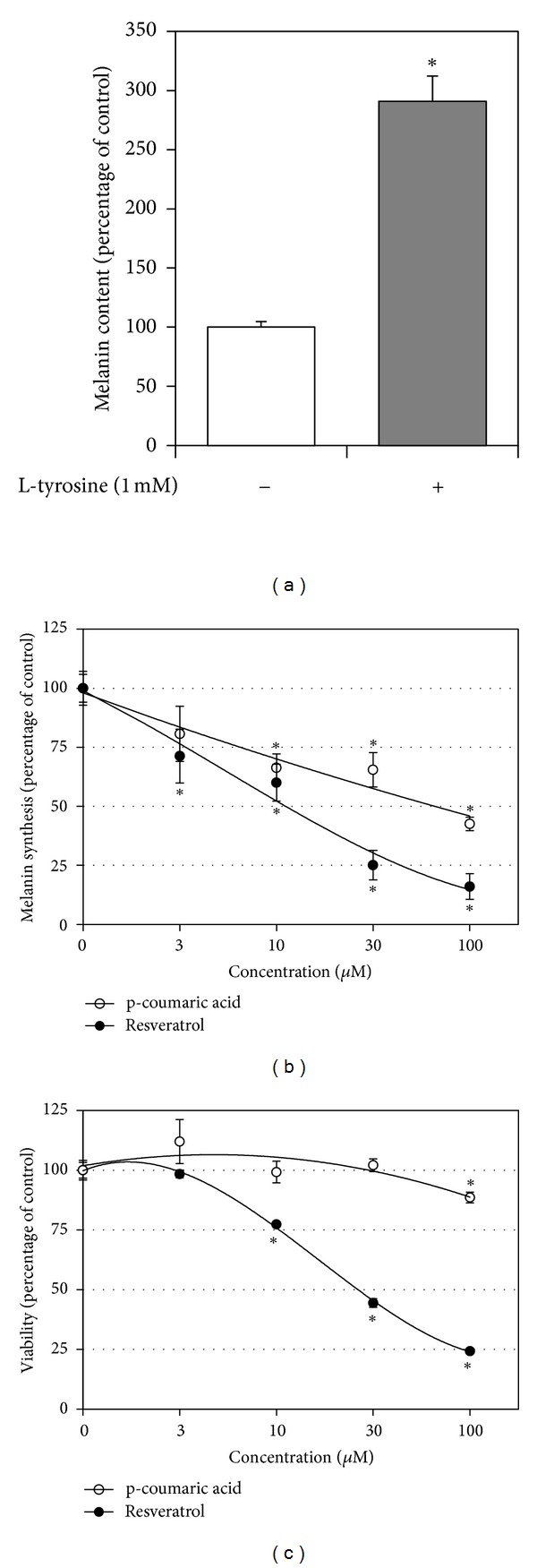
Effects of resveratrol and p-coumaric acid on melanin synthesis in HEMs. HEMs were repeatedly treated with 1.0 mM L-tyrosine (substrate) in the absence or presence of test material (inhibitor) at indicated concentrations for 6 days. Effect of L-tyrosine on intracellular melanin content was determined (a). The effects of resveratrol or p-coumaric acid on cellular melanin synthesis stimulated by L-tyrosine (b) and cell viability (c) were examined. Data are presented as percentages of vehicle controls (means ± SE, *n* = 3). *P < 0.05 versus vehicle control.

The bioactivities of resveratrol and p-coumaric acid were compared in HEMs. Cells were treated with 1.0 mM L-tyrosine (substrate) in the absence or presence of test material (inhibitor) at the indicated concentrations. This treatment was then repeated every other day for 6 days. As shown in Figure 7(a), treatment of HEMs with L-tyrosine increased intracellular melanin contents, and this melanin synthesis was dose dependently attenuated by resveratrol and p-coumaric acid (Figure 7(b)). Resveratrol was found to be slightly more effective than p-coumaric acid at inhibiting melanin synthesis. Repeated treatment of resveratrol above 10 *µ*M caused cytotoxicity whereas p-coumaric acid had only a little cytotoxic effect at 100 *µ*M (Figure 7(c)). 

## 4. Discussion

The present study shows that resveratrol is a highly potent and selective inhibitor of human TYR. A literature search indicated that opinions differ regarding the effects of resveratrol on TYR activity. Discrepancies between experimental results seem to be associated with different enzyme sources (human, murine, or mushroom). 

Resveratrol is oxidized by mushroom TYR and becomes a suicide inhibitor [26–28]. However its potency as a mushroom TYR inhibitor was determined to be very low (Figure 5(c)) compared to its potency as a human or murine TYR inhibitor (Figures 5(a) and 5(b)).

Kim et al. [29] reported that resveratrol inhibited murine TYR weakly (32.7% inhibition at 100 *µ*M or 22.8 *µ*g mL^−1^), but Yanagihara et al. [30] reported that it is a potent inhibitor (IC50 = 10.1 *µ*M = 2.3 *µ*g mL^−1^). Only the latter study showed a dose-response curve and is, though, more reliable. As observed in the present study, resveratrol produces insoluble precipitate at high concentrations, making the assay inaccurate. Thus, data from the assay at a single high concentration may be erratic. In the present study, the IC50 value of resveratrol against murine TYR was estimated to be 4.3 *µ*M (1.0 *µ*g mL^−1^) from a dose-response curve at low concentration range unaffected by solubility problem (Figure 5(b)).

Newton et al. [31] reported that resveratrol inhibited human TYR activity by ca. 75% at 20 *µ*g mL^−1^ in the assay using the lysates of HEMs. In our assay using the lysates of HEK293-TYR cells and HEMs, resveratrol was observed to inhibit human TYR activity by 80~90% at 10~30 *µ*g mL^−1^(Figures 5(a) and 6(c)). The IC50 values of resveratrol against human TYR activity were determined to be 1.7 *µ*M (0.39 *µ*g mL^−1^) with the lysates of HEK293-TYR cells (Figure 5(a)) and 5.3 *µ*M (1.2 *µ*g mL^−1^) with the lysates of HEMs (Figure 6(c)). Thus, the data from these studies consistently indicate that resveratrol is a potent inhibitor against human TYR.

Resveratrol has been previously shown to inhibit melanin synthesis in murine melanoma B16/F10 cells [28, 30] and HEMs [31, 32]. Based on our observation that resveratrol inhibits murine and human TYR potently, we are of the opinion that resveratrol might attenuate melanin synthesis through inhibition of TYR activity. Of course, other mechanisms could be involved in the inhibition of melanin synthesis by resveratrol. For example, resveratrol may regulate the posttranslational maturation of melanogenic enzymes in HEMs [31]. 

Previously, we have found that p-coumaric acid is a much stronger inhibitor of human TYR than kojic acid, arbutin [18], and various phenylpropanoids [19]. In the present study, resveratrol was found to be an even more potent inhibitor of human TYR than p-coumaric acid. In our bioassay using HEMs, resveratrol was also found to reduce melanin synthesis more effectively than p-coumaric acid (Figure 7(b)). However, resveratrol appeared to be slightly more cytotoxic than p-coumaric acid (Figure 7(c)). The mechanism of this cytotoxic effect of resveratrol is currently unclear but it could be an artifact due to culture conditions, because resveratrol is rapidly oxidized in carbonate-containing culture medium producing hydrogen peroxide [33]. Further studies are needed to evaluate the comparative efficacies and safeties of these two naturally occurring human TYR inhibitors *in vivo*. 

In the present study, resveratrol was found to be the main active constituent of VVC (Figure 2), the ethanolic extract of which inhibited human TYR activity potently (Figure 1). VVC is the dried stems of the grape tree (*Vitis vinifera* L.) belonging to Vitaceae. The polyphenolic composition and bioactivities of grape seed extracts have been extensively studied [34]. In contrast, grape stems have been barely investigated in this context. The present study shows that VVC is a useful source of resveratrol (Figure 4). Because resveratrol was found to be a potent inhibitor of human TYR and cellular melanogenesis, extracts of VVC containing resveratrol would be useful as skin whitening agents. The extract of Mori Ramulus (twigs of *Morus alba* L.) was also considered to be potentially useful as a skin whitening agent because it inhibited both mushroom and human TYR strongly (Figure 1). Indeed, previous studies have demonstrated its inhibitory effects against skin pigmentation in animal models [35]. Oxyresveratrol and mulberroside A have been proposed as active constituents [35, 36]. Further studies are needed to compare the human skin whitening effects of VVC and Mori Ramulus extracts.

HEK293-TYR cells are a useful source of human TYR needed in screening assays for melanogenesis inhibitors or skin whitening agents. HEK293-TYR cells constitutively and robustly express catalytically active human TYR and proliferate rapidly in relatively inexpensive culture media. This is a considerable advantage because HEMs grow slowly in expensive media. In addition, as compared with HEMs that expressed all melanogenic enzymes, HEK293-TYR cells overexpressed TYR only (Figure 6(a)). Thus, the cell lysates of HEK293-TYR cells are more suitable for TYR-specific assays, and their use makes data interpretation more straightforward. Overall, using HEK293-TYR cells, we were able to test many samples in a reasonable time at low cost. 

## 5. Conclusion

The present study shows that resveratrol is the main active compound in VVC responsible for the inhibition of human TYR activity. The use of HEK293-TYR cells enabled effective screening of plant sources and allowed us to resolve previously controversial observations regarding the effects of resveratrol on TYR activity and melanin synthesis. Furthermore, resveratrol was found to reduce melanin synthesis in HEMs at subtoxic concentrations. The findings of this study suggest that resveratrol and resveratrol-containing extracts of VVC have potential as skin whitening agents.

## Figures and Tables

**Table 1:** Medicinal plants examined during this study.

Extract number	Medicinal plants	Catalogue number	Yield of extract (% dry weight)
1	Acanthopanacis Cortex	CA03-021	2.86
2	Adenophorae Radix	CA01-098	1.60
3	Akebiae Caulis	CA02-030	5.84
4	Alismatis Rhizoma	CA04-064	4.80
5	Angelicae Tenuissimae Radix	CA02-014	10.00
6	Arisaematis Rhizoma	CA03-005	2.60
7	Artemisiae Apiaceae Herba	CA03-074	1.14
8	Artemisiae Iwayomogii Herba	CA04-036	3.33
9	Aurantii Nobilis Pericarpium	CA03-068	19.69
10	Bambusae Caulis in Taeniam	CA03-055	3.17
11	Bambusae Folium	CA03-057	4.36
12	Benincasae Semen	CA04-010	7.12
13	Biotae Orientalis Folium	CA03-076	6.22
14	Biotae Orientalis Folium (Roasted)	CA03-077	6.84
15	Cartami Semen	CA04-086	12.74
16	Castaneae Semen	CA01-007	1.29
17	Chaenomelis Fructus	CA01-051	3.17
18	Chrisanthemi Sibirici Herba	CA01-017	3.53
19	Cirsii Radix	CA02-025	5.00
20	Cnidii Rhizoma	CA03-070	4.73
21	Crataegi Fructus	CA02-041	2.01
22	Cyperi Rhizoma	CA03-088	2.08
23	Cyperi Rhizoma	CA03-089	3.60
24	Dioscoreae Rhizoma	CA04-023	1.87
25	Dioscoreae Rhizoma (Roasted)	CA04-024	1.85
26	Eucommiae Cortex (Roasted)	CA01-041	6.54
27	Eucommiae Folium	CA01-039	2.63
28	Eucommiae Ramulus	CA01-040	3.01
29	Forsythiae Fructus	CA02-075	5.00
30	Gardeniae Fructus (Roasted)	CA03-078	15.14
31	Glycine Semen Nigra	CA04-098	4.50
32	Liriopis Tuber	CA03-007	2.90
33	Lycopi Herba	CA04-062	3.76
34	Machili Thunbergii Cortex	CA04-069	4.75
35	Massa Medicata Fermentata	CA03-019	1.10
36	Mori Ramulus	CA04-028	3.27
37	Nepeta Spica	CA03-095	2.38
38	Paeoniae Radix Alba (Roasted)	CA01-073	2.41
39	Paeoniae Radix Alba	CA02-034	4.67
40	Perillae Folium	CA02-062	2.47
41	Peucedani Japonici Radix	CA01-062	7.09
42	Peucedani Radix	CA03-049	4.57
43	Pini Pollen	CA02-063	9.70
44	Pini Ramulus	CA04-030	22.40
45	Platycodi Radix	CA04-005	17.60
46	Polygoni Avicularis Herba	CA04-074	2.96
47	PrunusHumilis Semen	CA02-085	18.00
48	Rehmanniae Radix Crudus (Fresh)	CA04-042	8.69
49	Rehmanniae Radix	CA01-008	7.00
50	Santali Alba Lignum	CA04-017	1.31
51	Selaginellae Herba	CA02-021	8.88
52	Vitis Viniferae Caulis	CA04-077	2.90

**Table 2:** NMR data of purified compound X (resveratrol).